# SPSED: A Signal Peptide Secretion Efficiency Database

**DOI:** 10.3389/fbioe.2021.819789

**Published:** 2022-01-18

**Authors:** Chong Peng, Yixue Guo, Shaodong Ren, Cen Li, Fufeng Liu, Fuping Lu

**Affiliations:** ^1^ Key Laboratory of Industrial Fermentation Microbiology, Education Ministry of China, Tianjin, China; ^2^ National Engineering Laboratory for Industrial Enzymes (NELIE), Tianjin, China; ^3^ Tianjin Key Laboratory of Industrial Microbiology, Tianjin, China; ^4^ College of Biotechnology, Tianjin University of Science and Technology, Tianjin, China

**Keywords:** signal peptide, recombinant protein, secretion efficiency, database, bacteria

## Introduction

Signal peptides (SPs) are short amino acid sequences that direct the linked proteins into the secretory pathway. SPs are found in the N-terminus of proteins in virtually all organisms. Signal peptidases will remove signal peptides after the protein translocation. Signal peptides are usually 16–30 amino acids long and consist of a positively charged n-region, a hydrophobic h-region, and a c-region. The c-region contains the signal peptidase recognition site (von Heijne, 1990; 1998). Signal peptides are important in diverse fields that range from protein secretion mechanisms to disease diagnosis, especially in recombinant protein production (Freudl, 2018; Owji et al., 2018). For industrial enzymes production in bacterial cell factories, secreting the synthesized target proteins by the guidance of signal peptides will provide active and stable enzymes and a cost-effective downstream recovery process. In practice, different signal peptides show considerable differences in their ability to drive the secretion of the target protein. The optimum signal peptide for each recombinant protein is not consistent. The optimum signal peptide for one protein secretion could be inefficient for other proteins and *vice versa*. Systematic screening of a high-capacity signal peptide library has proven to be a powerful method to identify the optimal signal peptide for a target protein (Brockmeier et al., 2006; Mathiesen et al., 2009; Peng et al., 2019).

Admittedly, it is a time-consuming and labor-intensive job for researchers to pick out the optimum signal peptide for the target protein by experimental method. However, *in silico* identification of the best-performing signal peptide for a given protein is still not easy to implement. It remains unclear how the signal peptides influence the secretion efficiency of the recombinant proteins. Researchers can only get a partial understanding of this phenomenon by the non-integrated and fragmented data in the relevant literature. Two existing signal peptide databases, SPdb (Choo et al., 2005) and Signal Peptide Website (available at http://www.signalpeptide.com/) only contain signal peptide sequence but provide no information about signal peptide secretion capacity. A comprehensive collection of signal peptide secretion efficiency data is urgently needed to provide a reference for good-performing signal peptide selection in recombinant protein production. Herein, data about signal peptide secretion efficiency for specific target proteins were manually collected and a Signal Peptide Secretion Efficiency Database (SPSED) was constructed. SPSED is more focused on the signal peptide secretion efficiency for specific target proteins. SPSED is freely available at http://www.spsed.com/ with all major browsers supported. The database provides a user-friendly interface for browsing, searching, and downloading of SPSED records. Users can also BLAST a query sequence against SPSED to find a homologous secreted protein or signal peptide. We believe that SPSED is a valuable resource for recombinant protein production and researches in the mechanism of signal peptide secretion.

## Data Retrieval

Screening of a signal peptide library fused to the secretion target is an effective method to optimize the export of target protein. The signal peptide secretion efficiency data for alkaline active xylanase (Zhang et al., 2016), alkaline protease (Liu et al., 2019), aminopeptidase (Guan et al., 2016), subtilisin BPN’ (Degering et al., 2010), cutinase (Brockmeier et al., 2006; Hemmerich et al., 2016), natto phytase (Tsuji et al., 2015), nattokinase (Cai et al., 2016), nuclease (Mathiesen et al., 2009), and *α*-amylase (Fu et al., 2018) stored in SPSED are obtained by this method. Brockmeier et al. (2006) constructed a signal peptide library containing 173 predicted SPs from *Bacillus subtilis* 168. Cutinase from *Fusarium solani pisi* was used as the reporter protein. *B. subtilis* TEB1030 was used as the expression host. The screening revealed a dramatic difference in lipolytic activity of the culture supernatants. In this experiment, the metagenomic esterase EstCL1 was also used as the target protein with a subset of SPs in the library. Intriguingly, there was no correlation between the signal peptide secretion capacity for cutinase and esterase (Brockmeier et al., 2006). The comprehensive analysis of *Lactobacillus plantarum* signal peptide functionality reconfirmed the above conclusion. In this experiment, a signal peptide library containing 76 predicted signal peptides from *L. plantarum* WCFS1 was constructed. Signal peptides in the library showed considerable variation in terms of their performance to drive secretion of staphylococcal nuclease (NucA). To further test the signal peptides’ general usefulness, a selected set of SPs were used to direct the secretion of lactobacillal amylase (AmyA). Signal peptides’ secretion effect on AmyA and NucA were not consistent (Mathiesen et al., 2009). An optimal matching between the SP and the mature part of the target protein is essential for efficient protein secretion.

We retrieved articles that optimized protein secretion by signal peptides screening in the PubMed database, Google Scholar, and CNKI (China National Knowledge Infrastructure). We took the target proteins in the articles as objects and extracted the protein yield guided by different signal peptides manually from the articles. The nucleotide sequences and amino acid sequences of target proteins were then extracted from UniProt (Bateman et al., 2019) and GenBank database (Benson et al., 2013). The types and sources of SPs were obtained from the original articles. We got the signal peptides sequences directly if they were provided in the articles. If, on the other hand, the sequences of the signal peptides were not provided in the articles, we first downloaded the sequence of proteins from which the signal peptides come and then intercepted the SPs sequences according to SPs length or by signal peptide prediction server SingalP (Armenteros et al., 2019). Based on the sequences, we calculated the charge and hydrophobicity of the signal peptides and then drew the hydrophobicity plots.

## Database Description

**FIGURE 1:**
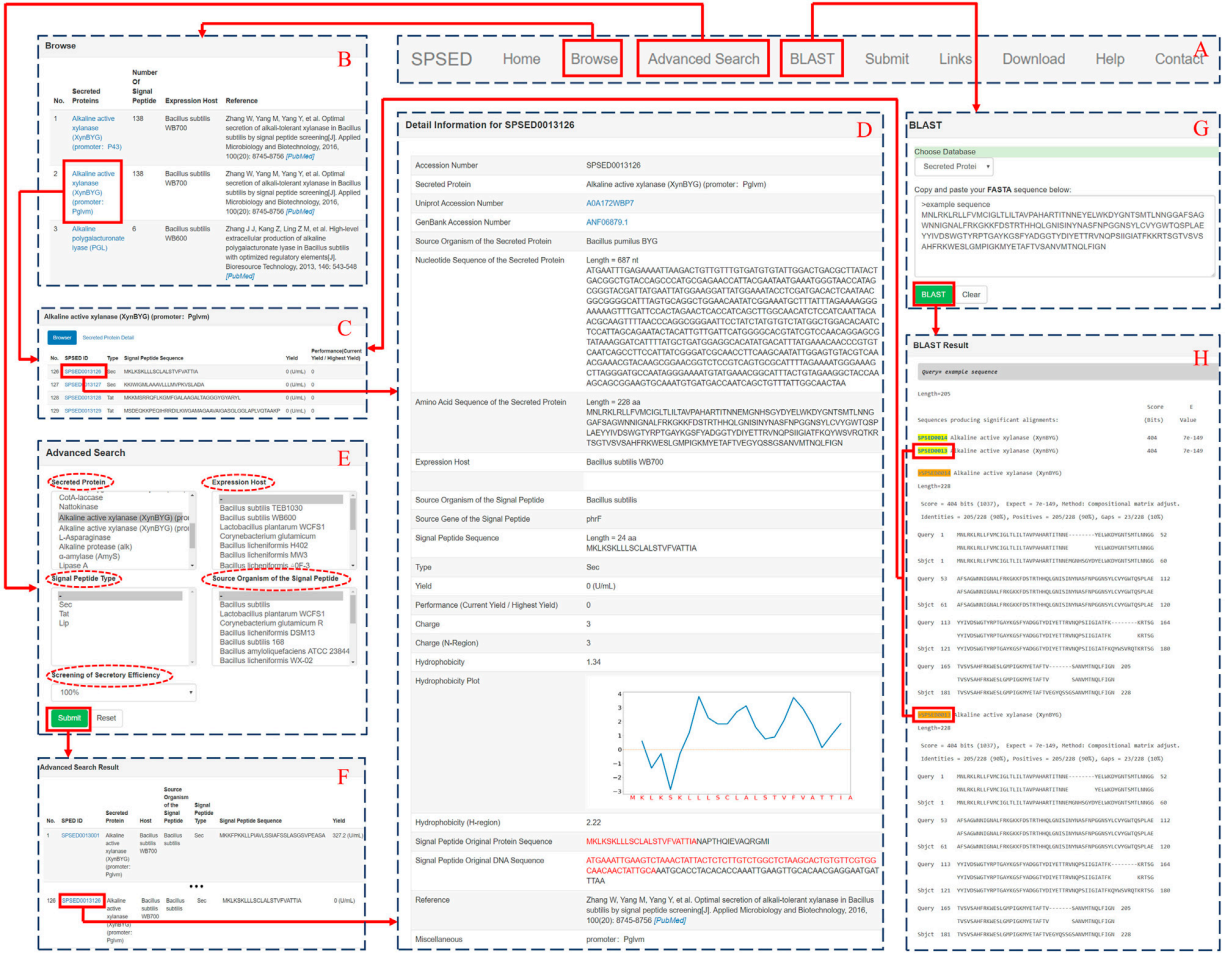
A screenshot of the SPSED database interface, which can illustrate the relationship among the main pages. **(A)** The global navigation bar which is located at the top of every page. **(B)** The browse page of the SPSED database. **(C)** Database browse interface for accessing the detailed information of target proteins and signal peptides. **(D)** A representative view of the record in SPSED. **(E)** The advanced search page of SPSED. **(F)** The advanced search result page of the database. **(G)** The BLAST search page of SPSED. **(H)** The BLAST result page of SPSED.

SPSED is built using SQLite allowing rapid retrieval of data and making resources easy to maintain. Figure 1 shows a snapshot of the SPSED database interface. The global navigation bar is located at the top of every page to enable the quick switch between different pages (Figure 1A). One entry in the database corresponds to the secretion yield of a specific target protein with the guidance of a specific signal peptide. We assigned a unique SPSED identification number for each record. The nucleotide sequence, amino acid sequence, UniProt link, GenBank link, and expression host of the secreted target protein have been displayed in both the ‘Secreted Protein Detail’ page and the ‘Detail Information’ page. The signal peptide source, type, sequence, DNA, and protein sequence of signal peptide original protein have been provided. We used the ratio of current yield to the highest yield to represent the secretion performance of each signal peptide. We also calculated the hydrophobicity of the whole signal peptide and the hydrophobicity of the h-region with values according to the Kyte-Doolittle hydrophobic scale (Kyte and Doolittle, 1982). The hydrophobicity plot of the whole signal peptide sequence is given on the signal peptide detail page. The charge of the whole signal peptide and the charge of the n-region is also calculated and provided in the database. Users can browse the secreted proteins and the secreted enzyme yield driven by different signal peptides (Figures 1B–D).

The database also provides an “Advanced Search” page for a customizable search of SPSED records. Users can select the options listed in the five select boxes named “Secreted Protein,” “Expression Host,” “Signal Peptide Type,” “Source Organism of the Signal Peptide,” and “Screening of Secretory Efficiency” to filter out the records they are interested in (Figures 1E,F). Besides, we have installed the BLAST (Altschul et al., 1997) program locally. When the protein or signal peptide of interest is not in SPSED, users can BLAST the query sequence against our database to find a homologous secreted protein or signal peptide. The query amino acid sequence is required to be pasted in the textbox in fasta format. When the alignment is ready, a BLAST result page with links to the database records is provided (Figures 1G,H). Users are encouraged to submit new data via the ‘Submit’ page. The submitted data will be manually revised and incorporated into the release of the SPSED database. The “Links” page provides a list of tools for signal peptide prediction and web resources related to protein secretion. Target proteins yield that is driven by different signal peptides are packaged by the expression hosts and the target proteins. The compressed data are presented on the ‘Download’ page for batch downloading. The SPSED database is available online at http://www.spsed.com/and requires no registration.

## Conclusion and Perspectives

In conclusion, we have developed a signal peptide secretion efficiency database SPSED. This database is, to our knowledge, the first attempt to provide the yield of industrial enzymes with the guidance of different signal peptides, which can reflect the signal peptide secretion capacity for the target protein. In the current version of SPSED, 1025 signal peptide secretion efficiency data collected from 20 experiments are included. SPSED has been experiencing slow and linear database growth because all records in the SPSED database are manually curated. Manual selection is required in literature mining to pick out articles that report detailed enzyme activity data. Besides, the target protein, expression host, signal peptide, and enzyme activity data in the literature also need to be collected manually. It is now difficult to develop an automated process to gather data for SPSED in batch. Encouraging users to submit their signal peptide secretion efficiency data could be a potential way for a faster inclusion of records. Anyway, the maintenance and revision of the SPSED database will keep on going. We believe that SPSED will facilitate the production of enzymes and studies on the mechanism of signal peptide secretion. Biotechnologists who work on recombinant protein production can pick out a good-performing signal peptide for their target protein from this database. Microbiologists can also investigate the mechanism of efficient protein secretion by analyzing data in SPSED, which is worth exploring.

## Data Availability

The original contributions presented in the study are included in the article, further inquiries can be directed to the corresponding authors.

## References

[B1] Almagro Armenteros J. J. Tsirigos K. D. Sønderby C. K. Petersen T. N. Winther O. Brunak S. (2019). SignalP 5.0 Improves Signal Peptide Predictions Using Deep Neural Networks. Nat. Biotechnol. 37 (4), 420–423. 10.1038/s41587-019-0036-z 30778233

[B2] Altschul S. Madden T. L. Schaffer A. A. Zhang J. Zhang Z. Miller W. (1997). Gapped BLAST and PSI-BLAST: a New Generation of Protein Database Search Programs. Nucleic Acids Res. 25 (17), 3389–3402. 10.1093/nar/25.17.3389 9254694 PMC146917

[B3] Bateman A. Martin M.-J. Orchard S. Magrane M. Alpi E. Bely B. (2019). UniProt: a Worldwide Hub of Protein Knowledge. Nucleic Acids Res. 47 (D1), D506–D515. 10.1093/nar/gky1049 30395287 PMC6323992

[B4] Benson D. A. Cavanaugh M. Clark K. Karsch-Mizrachi I. Lipman D. J. Ostell J. (2013). GenBank. Nucleic Acids Res. 41 (D1), D36–D42. 10.1093/nar/gks1195 27899564 PMC5210553

[B5] Brockmeier U. Caspers M. Freudl R. Jockwer A. Noll T. Eggert T. (2006). Systematic Screening of All Signal Peptides from Bacillus Subtilis: A Powerful Strategy in Optimizing Heterologous Protein Secretion in Gram-Positive Bacteria. J. Mol. Biol. 362 (3), 393–402. 10.1016/j.jmb.2006.07.034 16930615

[B6] Cai D. Wei X. Qiu Y. Chen Y. Chen J. Wen Z. (2016). High-level Expression of Nattokinase in Bacillus Licheniformis by Manipulating Signal Peptide and Signal Peptidase. J. Appl. Microbiol. 121 (3), 704–712. 10.1111/jam.13175 27159567

[B7] Choo K. H. Tan T. W. Ranganathan S. (2005). SPdb - a Signal Peptide Database. BMC Bioinformatics 6, 249. 10.1186/1471-2105-6-249 16221310 PMC1276010

[B8] Degering C. Eggert T. Puls M. Bongaerts J. Evers S. Maurer K.-H. (2010). Optimization of Protease Secretion in Bacillus Subtilis and Bacillus Licheniformis by Screening of Homologous and Heterologous Signal Peptides. Appl. Environ. Microbiol. 76 (19), 6370–6376. 10.1128/aem.01146-10 20709850 PMC2950444

[B9] Freudl R. (2018). Signal Peptides for Recombinant Protein Secretion in Bacterial Expression Systems. Microb. Cel Fact 17 (1), 52. 10.1186/s12934-018-0901-3 PMC5875014 29598818

[B10] Fu G. Liu J. Li J. Zhu B. Zhang D. (2018). Systematic Screening of Optimal Signal Peptides for Secretory Production of Heterologous Proteins in Bacillus Subtilis. J. Agric. Food Chem. 66 (50), 13141–13151. 10.1021/acs.jafc.8b04183 30463403

[B11] Guan C. Cui W. Cheng J. Liu R. Liu Z. Zhou L. (2016). Construction of a Highly Active Secretory Expression System via an Engineered Dual Promoter and a Highly Efficient Signal Peptide in Bacillus Subtilis. New Biotechnol. 33 (3), 372–379. 10.1016/j.nbt.2016.01.005 26820123

[B12] Hemmerich J. Rohe P. Kleine B. Jurischka S. Wiechert W. Freudl R. (2016). Use of a Sec Signal Peptide Library from Bacillus Subtilis for the Optimization of Cutinase Secretion in Corynebacterium Glutamicum. Microb. Cel Fact 15 (1), 208. 10.1186/s12934-016-0604-6 PMC5142396 27927208

[B13] Kyte J. Doolittle R. F. (1982). A Simple Method for Displaying the Hydropathic Character of a Protein. J. Mol. Biol. 157 (1), 105–132. 10.1016/0022-2836(82)90515-0 7108955

[B14] Liu Y. Shi C. Li D. Chen X. Li J. Zhang Y. (2019). Engineering a Highly Efficient Expression System to Produce BcaPRO Protease in Bacillus Subtilis by an Optimized Promoter and Signal Peptide. Int. J. Biol. macromolecules 138, 903–911. 10.1016/j.ijbiomac.2019.07.175 31356949

[B15] Mathiesen G. Sveen A. Brurberg M. Fredriksen L. Axelsson L. Eijsink V. G. (2009). Genome-wide Analysis of Signal Peptide Functionality in Lactobacillus Plantarum WCFS1. BMC Genomics 10 (1), 425. 10.1186/1471-2164-10-425 19744343 PMC2748100

[B16] Owji H. Nezafat N. Negahdaripour M. Hajiebrahimi A. Ghasemi Y. (2018). A Comprehensive Review of Signal Peptides: Structure, Roles, and Applications. Eur. J. Cel Biol. 97 (6), 422–441. 10.1016/j.ejcb.2018.06.003 29958716

[B17] Peng C. Shi C. Cao X. Li Y. Liu F. Lu F. (2019). Factors Influencing Recombinant Protein Secretion Efficiency in Gram-Positive Bacteria: Signal Peptide and beyond. Front. Bioeng. Biotechnol. 7, 139. 10.3389/fbioe.2019.00139 31245367 PMC6579943

[B18] Tsuji S. Tanaka K. Takenaka S. Yoshida K.-i. (2015). Enhanced Secretion of Natto Phytase by Bacillus Subtilis. Biosci. Biotechnol. Biochem. 79 (11), 1906–1914. 10.1080/09168451.2015.1046366 26023739

[B19] von Heijne G. (1998). Life and Death of a Signal Peptide. Nature 396 (6707), 111–113. 10.1038/24036 9823886

[B20] von Heijne G. (1990). The Signal Peptide. J. Membrain Biol. 115 (3), 195–201. 10.1007/bf01868635 2197415

[B21] Zhang W. Yang M. Yang Y. Zhan J. Zhou Y. Zhao X. (2016). Optimal Secretion of Alkali-Tolerant Xylanase in Bacillus Subtilis by Signal Peptide Screening. Appl. Microbiol. Biotechnol. 100 (20), 8745–8756. 10.1007/s00253-016-7615-4 27225471

